# Colouterine and Jejunouterine Fistula Secondary to Chronic Diverticulitis

**DOI:** 10.1155/2021/5543505

**Published:** 2021-04-01

**Authors:** Ilias Galanis, Georgios Floros, Christophoros Theodoropoulos, Myriam Metaxa, Panagiotis Theodoropoulos, Panagiotis Tsintavis, Dimitrios Bartziotas, Georgios Giannos, Georgios Stylianidis, Georgios Papadopoulos

**Affiliations:** Department of Surgery, Evaggelismos General Hospital, Athens, Greece

## Abstract

Fistulae between the colon or the small intestine and the uterus are extremely rare as the uterus is a thick, muscular organ. Here, we present the case of a 74-year-old female presenting to our surgical department because of fecal vaginal discharge for the past few months, which proved to be caused by a combined colouterine and jejunouterine fistula due to chronic diverticulitis. Total abdominal hysterectomy with bilateral oophorectomy with en bloc resection of part of the jejunum and the sigmoid colon and primary anastomoses were performed. This case represents an unusual type of diverticulitis complication and aims to point out the diagnostic and therapeutic issues of such a rare medical condition.

## 1. Introduction

Diverticulosis is one of the most common colonic pathologies. It affects, mostly, those who are living in developed countries, and its incidence is well known to increase with age. However, the incidence of diverticular disease has, recently, been increasing in Third-World countries [1]. Diverticulitis is the result of infection and inflammation of diverticula. The complications of diverticulitis are stricture, bleeding, perforation, fistula formation, and death [2]. Fistulisation is defined as a communication between two surfaces getting in contact [3]. Fistulae constitute up to 20% of complications. The formation of the fistula results from a local inflammatory process which decompresses by perforating into an adjacent tissue. The most common type of fistulae due to diverticulitis is colovesical fistulae, with colovaginal fistulae being the second most common. Colouterine, as well as jejunouterine, fistulae are very rare because the uterus is a very thick and muscular organ, preventing any benign or malignant disease from invading it [4]. Except for diverticulitis, malignant disease, spontaneous rupture of a gravid uterus, and obstetric trauma such as curettage and radiation therapy may result in a fistula between the intestine and the uterus [5]. A colouterine fistula was, first, described by Lejemtel in 1909, and the first report of the colouterine fistula related to diverticulitis was given by Noecker in 1929 [1]. Since then, only very few cases have been reported in the literature, indicating the rarity of this condition.

## 2. Case

**Figure 1:**
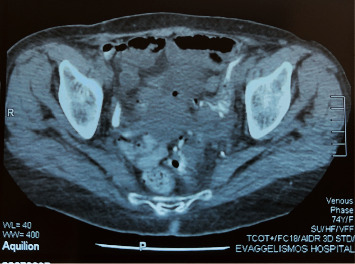
CT scan showing findings of chronic diverticulitis.

**Figure 2:**
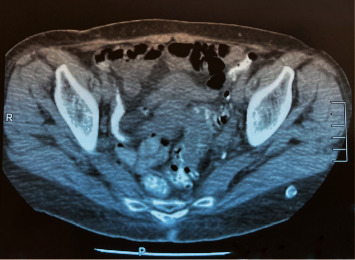
CT scan showing findings of chronic diverticulitis.

**Figure 3:**
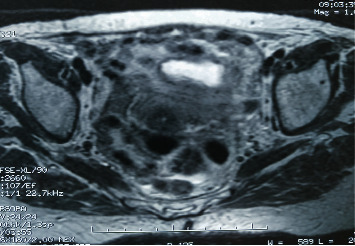
MRI revealing the colouterine fistula.

**Figure 4:**
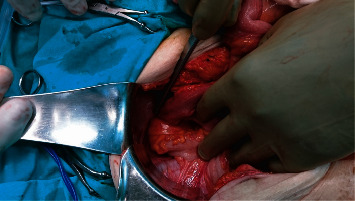
Colouterine and jejunouterine fistulae discovered intraoperatively.

**Figure 5:**
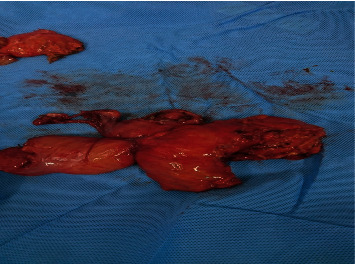
En bloc resection of the uterus with part of the sigmoid colon and jejunum.

**Figure 6:**
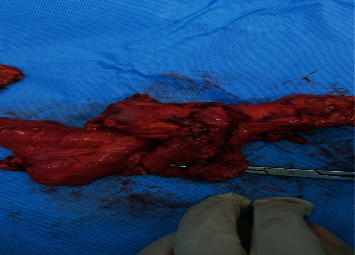
En bloc resection of the uterus with part of the sigmoid colon and jejunum.

A 74-year-old female was referred to our department of surgery with malodorous fecal vaginal discharge that had lasted several weeks. Medical history included multiple episodes of acute diverticulitis and dementia. She had no abdominal pain or fever. Upon admission, her laboratory tests were within the normal range. Pelvic examination revealed an atrophied vagina and malodorous yellowish vaginal discharge. A colovaginal fistula, secondary to diverticular disease, was suspected as it is the most common type of fistula between the gastrointestinal and female reproductive system. Cultures of the vaginal discharge were sent. Nil per os, total parenteral nutrition, and intravenous antibiotic therapy with ciprofloxacin and metronidazole were applied to the patient until a definitive diagnosis would be made. An abdominal computed tomography (CT) showed multiple diverticula and findings indicative of chronic diverticulitis, such as thickening of the wall of the sigmoid colon and pericolic fat stranding (Figures 1 and 2). A colonoscopy was performed, revealing diverticula of the sigmoid colon. Magnetic resonance imaging (MRI) revealed a fistula between the sigmoid colon and the uterine fundus and air bubbles in the uterine cavity and the vagina (Figure 3). Surgical management was decided. Intraoperative findings included a colouterine fistula side to side to a jejunouterine fistula as well (Figure 4). Inflammatory adhesions of the sigmoid colon and the jejunum to the wall of the uterus, as a result of the repeated episodes of diverticulitis, were found, explaining the presence of the fistulae, as well as adhesive lesions between the urinary bladder and uterus. Taking in mind the age of the patient and the intraoperative findings, we performed a total abdominal hysterectomy with bilateral oophorectomy and en bloc resection of the defective part of the sigmoid colon and jejunum (Figures 5 and 6). Primary jejunojejunal and colocolonic anastomoses were performed. During surgery, a bladder injury was recognized and repaired immediately. Postoperatively, the patient had a fever because of an urinary tract infection which was treated with antibiotics. Rest of the postoperative course was uneventful, and the patient was discharged on the 21^st^ postoperative day.

## 3. Discussion

Fistula creation between the gastrointestinal tract and the uterus is rare. The fistula is usually found between the fundus of the uterus and loops of the sigmoid colon or small bowel [6]. In cases of diverticulitis, the inflammatory adherence of the bowel wall to the uterus may result in fistula formation, especially in older people whose uterine wall becomes thinner and mucosa atrophic. Clinical manifestations of a colouterine or a jejunouterine fistula may vary, but typical symptoms include malodorous fecal or purulent vaginal discharge for weeks or months. Fever and recurrent abdominal pain may, also, be present, in case of diverticulitis, but they may be absent in case of a chronic inflammation, as in our case. A palpable abdominal mass may be reported, especially if an abscess has been described [7].

Many imaging modalities have been suggested for establishing the diagnosis of a colouterine or jejunouterine fistula. Vaginal or cervical cultures will determine the source of vaginal discharge and will be useful for selecting the appropriate antibiotic therapy. The imaging modality for diverticular fistulae has been contrast radiology, either rectally or vaginally. Computed tomography (CT) may reveal evidence of diverticulitis and communication between the uterus and the colon, but it may fail to demonstrate the fistulous tract. A multidetector computed tomography (MDCT) has been used to provide better visualisation of the fistula, but it is associated with great radiation. Magnetic resonance imaging (MRI) is a more accurate diagnostic tool, able to identify fistulae, and demonstrate the degree of pericolic inflammation, especially in T1-weighted images. In our case, MRI was the effective modality for diagnosis as it was the only imaging technique indicating the colouterine fistula. The charcoal test depends on demonstrating the passage of orally administered activated charcoal from the cervical os at pelvic examination on the next day. A colonoscopy may be useful in the diagnosis of diverticulosis, but it is not very helpful in the detection of fistulae. Finally, endovaginal ultrasonography and sonohysterography using a contrast medium could be used to visualise both the fistula tract and the uterine wall and colon [1, 8].

Surgical management is the treatment of choice, in most cases. Many surgical approaches have been suggested. Hartmann procedure with hysterectomy, transverse colostomy followed by hysterectomy and closure of the fistula without bowel resection, and an en bloc resection of the uterus and the colon or small bowel are several examples [9]. Hysterectomy may be necessary if malignancy is suspected, but it is not mandatory in case of benign disease [7]. When there is severe local inflammation, a two-stage procedure with reanastomosis at a later time is preferable. In our case, because of the age of the patient and the fact that there was no acute infection, we performed a total abdominal hysterectomy with bilateral oophorectomy and en bloc resection of the sigmoid colon and part of the jejunum and primary anastomoses of the intestines. Laparoscopic approach may, also, be used [2]. Conservative treatment has been suggested with antibiotics and drainage of any abscesses, but it is used, mainly, in patients with major contradictions for surgery [1]. Prognosis for patients with fistulae secondary to diverticulitis is excellent after surgical treatment [9].

## 4. Conclusion

Fistulae between the uterus and the bowel are very rare complications of diverticulitis. They should be suspected in case of malodorous discharge from the vagina so that the appropriate diagnostic tests may be conducted and the best therapeutic options may be applied. Accurate diagnosis is the key to prompt treatment of this very rare condition.

## Data Availability

The data that support the findings of this case report are available from the corresponding author, I. Galanis, upon reasonable request.
